# An autoencoder-based snow drought index

**DOI:** 10.1038/s41598-023-47999-5

**Published:** 2023-11-24

**Authors:** Sinan Rasiya Koya, Kanak Kanti Kar, Shivendra Srivastava, Tsegaye Tadesse, Mark Svoboda, Tirthankar Roy

**Affiliations:** 1 Department of Civil and Environmental Engineering, University of Nebraska-Lincoln, Lincoln, USA; 2 National Drought Mitigation Center, University of Nebraska-Lincoln, Lincoln, USA

**Keywords:** Hydrology, Natural hazards

## Abstract

In several regions across the globe, snow has a significant impact on hydrology. The amounts of water that infiltrate the ground and flow as runoff are driven by the melting of snow. Therefore, it is crucial to study the magnitude and effect of snowmelt. Snow droughts, resulting from reduced snow storage, can drastically impact the water supplies in basins where snow predominates, such as in the western United States. Hence, it is important to detect the time and severity of snow droughts efficiently. We propose the Snow Drought Response Index or SnoDRI, a novel indicator that could be used to identify and quantify snow drought occurrences. Our index is calculated using cutting-edge ML algorithms from various snow-related variables. The self-supervised learning of an autoencoder is combined with mutual information in the model. In this study, we use Random Forests for feature extraction for SnoDRI and assess the importance of each variable. We use reanalysis data (NLDAS-2) from 1981 to 2021 for the Pacific United States to study the efficacy of the new snow drought index. We evaluate the index by confirming the coincidence of its interpretation and the actual snow drought incidents.

## Introduction

In many regions worldwide, snow has a vital contribution to drought occurrence^1^, evident from the role of snow in regional and global water resources and climate^2–4^. Recently, it has led to a broad discussion on the association between droughts and snow, along with the emergence of several studies focusing on “snow drought,” indicative of lower-than-normal snow conditions^1,5–9^. However, a consistent way of characterizing snow droughts is missing in these past studies, resulting in the absence of a solid framework to detect snow droughts.

Different authors defined snow droughts differently, making the analysis of these droughts conditioned upon and potentially sensitive to the definitions. For example, a recent study assessing the global snow drought hotspots and characteristics considered a snow drought event as a deficit of snow water equivalent (SWE)^1^. Another study argued that defining snow droughts just in terms of SWE might not be sufficient, and it referred to snow drought as a combination of general droughts and shortages in snow storage, reflecting both the lack of winter precipitation and SWE^5^. Subsequently, Hatchett & McEvoy (2018) defined different types of snow droughts based on the origination, persistence, and termination of below-normal snow accumulations. Later, several studies expressed snow droughts in terms of threshold percentiles, which is essentially subjective^8,10^. Some of these studies used the median SWE (of the cooler months in the entire time period) to identify snow droughts, whereas others used maximum SWE^8,10^. Although we need an index to study and predict snow droughts, the lack of coherence in the characterization of snow droughts potentially questions the reliability of a snow drought index based on strict definitions. Therefore, we need a framework to calculate a snow drought index independent of such definitions, which, at the same time, can also capture the signals of a snow drought, considering factors connected to snow accumulation and ablation.

To date, only a limited number of studies have been conducted on snow drought indices. The recently developed Standardized Snow Water Equivalent Index (SWEI) is obtained through the inverse standard normal distribution of probabilities associated with SWE integrated over three months^1^. Keyantash and Dracup^11^ developed the aggregated drought index (ADI) based on rainfall, evapotranspiration, streamflow, reservoir storage, soil moisture, and snow water content variables. Here, a principal component analysis (PCA) was employed to reduce the dimensionality and explain the key variabilities represented by the selected variables. Staudinger et al.^12^ developed an extension to the Standardized Precipitation Index (SPI) named Standardized Snow Melt and Rain Index (SMRI), where they used the sum of rainfall and snowmelt variables instead of precipitation. Qiu^13^ modified the standard Palmer Drought Severity Index (PDSI; Palmer, 1965^14^) by including degree day factor (DDF), an empirical threshold temperature-based snowmelt model, to account for snow processes. This modification improved the drought monitoring capabilities in several snow-dominated regions^13,15^. However, these indices often depend upon in-situ observations, which might not be readily available in many regions, and the problem is exacerbated in ungauged or sparsely gauged regions. This calls for an index that can leverage remote sensing datasets and bypass the need for extensive in-situ observation networks.

Merging and extracting necessary information on snow droughts from a wide range of variables present in remote sensing datasets can be challenging since not all variables are equally related to the formation of snow droughts. However, we need to identify important variables so that they can be merged to form one single index. Machine learning (ML)-based feature selection algorithms are promising in this regard since they can filter our variables based on their importance^16–19^. Thus, we can use ML techniques to infer the influence of hydroclimatic variables on snow droughts. Apart from this, information theory-based methods can manifest the relative influence of variables and their causal connections^20–22^. Mutual Information (MI), a measure of how much information about one random variable is contained in another random variable, has been widely applied in feature selection problems^23,24^.

In this study, we introduce a new snow drought index, the Snow Drought Response Index (SnoDRI), and a novel drought index estimation approach using a combination of ML techniques and MI. Our framework is advantageous for multiple reasons: (1) our index is not based on any specific variable, instead, it aggregates information from multiple variables, (2) we do not require any in-situ observations, thus enabling snow drought detection in ungauged basins, (3) our framework is explainable, i.e., it can provide insights into crucial features impacting the occurrence of snow drought. Our results show that SnoDRI could successfully detect the signals of a historical snow drought event.

## Methods

### Study area

**Figure 1:**
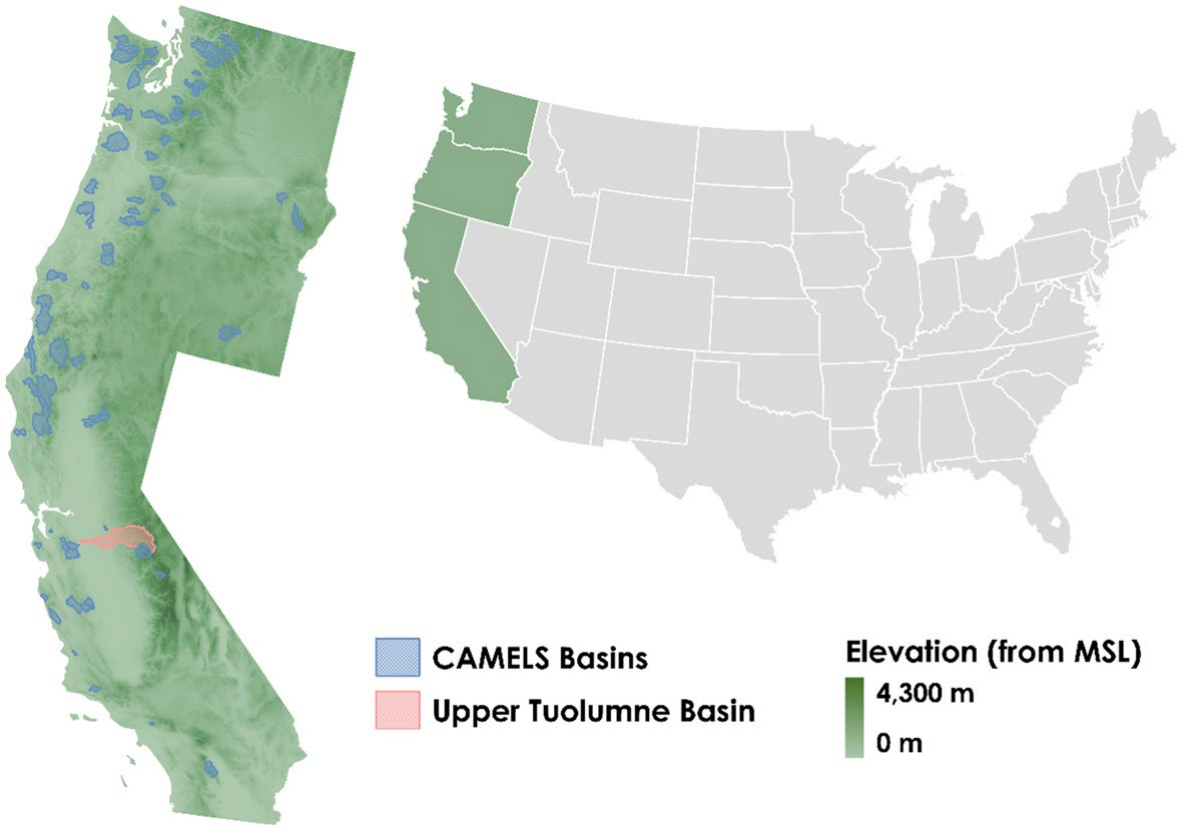
The Pacific Coast States of the United States showing the study basins. Created using QGIS 3.22.1-Białowieża. Reference: QGIS Development Team, 2023. QGIS Geographic Information System. Open Source Geospatial Foundation. URL http://qgis.org.

The Western United States is characterized as a snow drought hot spot where snow drought becomes widespread, intensified, and prolonged^1^. Snow plays a significant role in the hydrology of the Western United States. The maximum annual SWE accumulation in the region is 1176 mm (elevation 2835 m) at Mammoth Pass, California^25^. During spring or early summer, discharge in the high-elevation watersheds that drain through mountains contains a substantial amount of water as a result of warming snowpacks and snowmelt^26,27^. Recent events have shown that the water resources and management in this region are hugely influenced by snow droughts^5^. Therefore, this study focuses on three states (Pacific Coast States) in the western USA: Washington, California, and Oregon (Fig. 1). The highest elevation of this region ranged from 1,547 m in California to 520 m in Washington State. As a result of significant fluctuation in the elevation, the orographic precipitation and the variability in average annual maximum snow water accumulation are considerable.

To validate our results, we select a mountainous area situated in the upper Tuolumne River basin of the Sierra Nevada^28^. About 60% of water in Southern California is received from the Sierra Nevada snowpack^29^. As such, the snowpack plays a vital role in this region, which was confirmed by the impacts of below-normal snow conditions on water resources, ecosystems, and recreation^8^. Due to the mountainous region and varied elevation, individual climatological extreme events can produce a drastically different response in magnitude and spatial variability of the snowpack in this region^30^. Recently, 2014 and 2015 showed a lack of snow accumulation and winter precipitation, indicating snow drought events in the upper Tuolumne basin^5,8,9^. The historical precipitation trends in this region have shifted from snow to rain, resulting in more frequent droughts^10,31^. This shift has impacted headwater hydrology and downstream reservoir management of basins in the Sierra Nevada^32^.

### Data used for SnoDRI

#### NLDAS-2 variables

The North American Land Data Assimilation System (NLDAS) is a multi-institutional partnership project intended to develop land-surface model datasets from observations and reanalysis with quality control that is coherent across space and time^33^. NLDAS data is comprised of hourly data in gridded format with a spatial resolution of 0.125° × 0.125°. An improved version, NLDAS-2, was later developed by determining and rectifying existing errors in forcing data and models^34^. NLDAS-2 changed data sources and their inherent biases, upgraded the model along with recalibrated parameters, and increased the period of forcing data and simulations^34^. We use NLDAS-2 primary (File A) forcing data, which provides a total of eleven variables, given in Table 1. All these variables are spatially aggregated for interested basins and converted to monthly time series (cumulative variables-APCP, PEVAP, CAPE, DLWRF, and DSWRF- are added, others averaged). It is important to note that we do not use snowfall separately since there could be important information in the total precipitation. For example, rain-on-snow events significantly impact snow accumulation and melt^35^. We might inhibit the model from learning crucial information using only snowfall. Here, in our framework, the idea is to provide available data, which might or might not be important in drought stress formation, and let the model figure out the key information.

**Table 1:** List of variables considered for analysis.

**#**	Variables name	Abbreviat-ion	Reso-lution	Period	Sources
1	U wind component (m/s) at 10 m above the surface	UGRD	0.125° × 0.125°	1979–2020 Hourly	NLDAS 2
2	V wind component (m/s) at 10 m above the surface	VGRD			
3	Air temperature (K) at 2 m above the surface	TMP			
4	Specific humidity (kg/kg) at 2 m above the surface	SPFH			
5	Surface pressure (Pa)	PRES			
6	Surface downward longwave radiation (W/m^2)	DLWRF			
7	Surface downward shortwave radiation (W/m^2)	DSWRF			
8	Precipitation hourly total (kg/m^2)	APCP			
9	Fraction of total precipitation that is convective (no units)	CONVfrac			
10	Convective available potential energy (J/kg)	CAPE			
11	Potential evaporation (kg/m^2^)	PEVAP			
12	SWE	SWE	4 x 4 km	1982–2020 Daily	NSIDC
13	Discharge	Q	Point	1979–2020 Daily	USGS

#### Standardized precipitation index (SPI)

The Standardized Precipitation Index (SPI) is an index widely used for quantifying precipitation anomalies^36^. The SPI is obtained by mapping the actual probability distribution of precipitation to a normal distribution. A zero SPI indicates normal conditions. The positive values of SPI represent wet conditions, whereas the negative values represent dry conditions. The larger the negative value of SPI, the higher the severity of drought conditions. We calculate SPIs at three timescales (3, 4, and 6 months) and provide them as inputs for the SnoDRI because they provide a combined measure of precipitation over multiple time scales. The SPI at 3, 4, and 6 months timescales are chosen because the snow processes and the impact of reduced winter precipitation generally occur at these timescales. These SPIs can reflect the reduced snowpack and discharge from snowmelt. For the same reason, SPIs at longer timescales (such as SPI-12 and 60) are potentially unimportant for snow droughts. Therefore, we have also fed the model with SPI-12 and SPI-60 as a sanity check to see whether the model can discard irrelevant information, which is confirmed by our results (see Sect. “weights from mutual information”). The SPI calculation only requires precipitation; it smoothens the time series data and maps the actual distribution of precipitation to normal distribution. We use the basin-aggregated NLDAS-2 precipitation for computing SPIs.

#### Snow water equivalent

We use snow water equivalent (SWE) as an indicator variable for the snow drought case used for validation. SWE is the target variable for the Random Forest model (discussed in a later section) used in selecting the input features for our index. We obtained SWE data and snow depth from assimilated in-situ and modeled data over the conterminous US, Version 1^37,38^ from the National Snow and Ice Data Center (NSIDC). This data provides daily SWE and snow depth at a spatial resolution of 4 km x 4 km for the conterminous United States (CONUS). We collected the SWE data from 1982 to 2020, spatially aggregated them for the study basins, and converted them to monthly time series.

#### CAMELS basin shapefiles

Catchment Attributes and MEteorology for Large-sample Studies (CAMELS) provide the time series of meteorological forcings and attributes of 671 basins across the CONUS^39,40^. These basins are least affected by human actions^40^. The dataset contains different categories of basin attributes: topography, climate, streamflow, land cover, soil, and geology^40^. We use the basin shapefiles provided in the dataset and filtered basins (a total of 85) belonging to the Pacific Coast states. The gridded datasets are aggregated to the basin scale using these shapefiles.

#### Discharge

The discharge data for these basins were collected from the US Geological Survey’s (USGS) streamflow measurements provided in the CAMELS dataset. USGS collects, monitors, and analyzes existing resource conditions across the different sites in the US. USGS stations measure velocity through a current meter or acoustic Doppler current profiler. The discharge is computed by multiplying the cross-sectional area by the estimated velocity. For this study, daily data were obtained from 1979 through 2020. The daily flow records for USGS gage stations provide the value of mean discharge for each day of the water year^41^.

### SnoDRI framework

**Figure 2:**
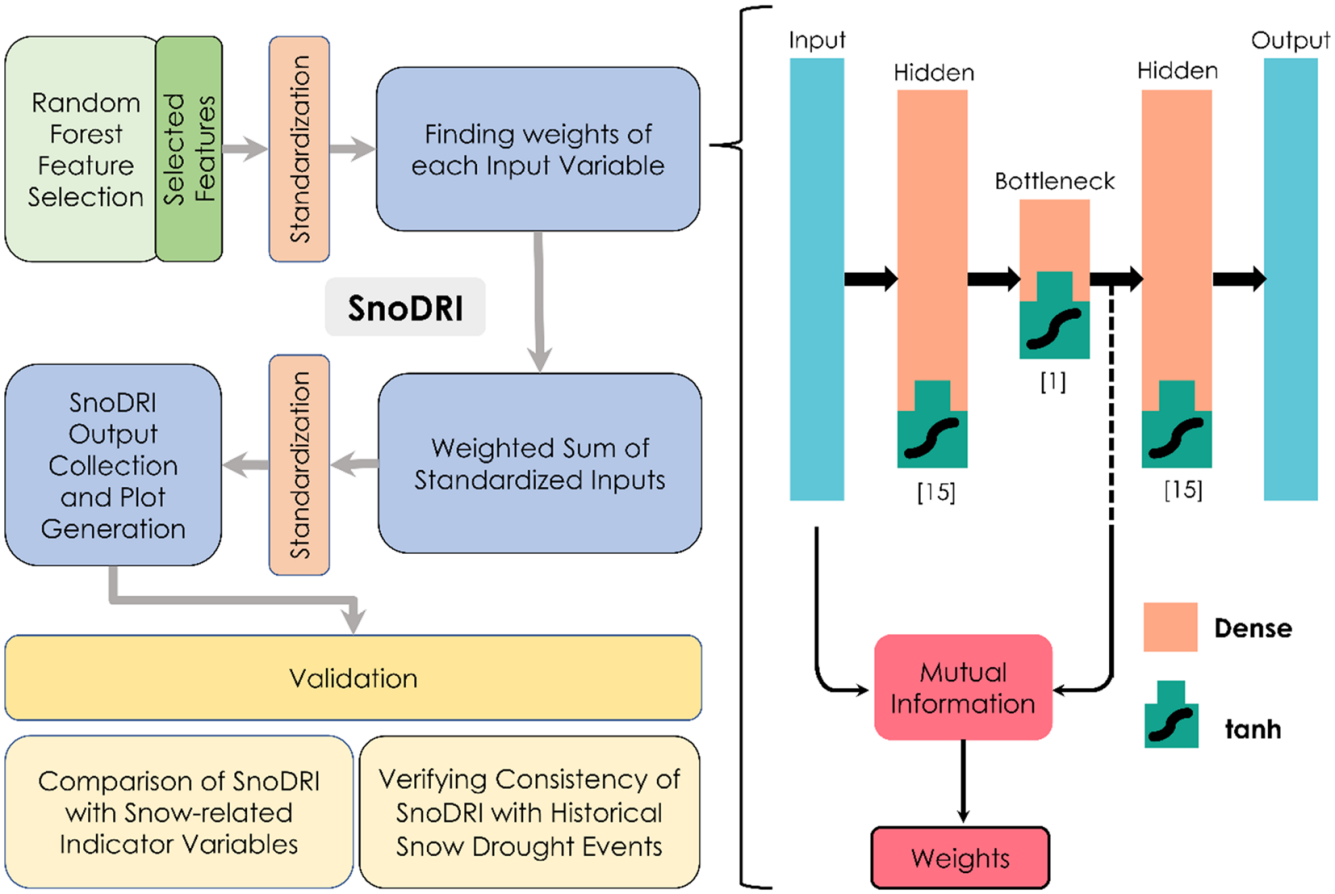
The methodology used for developing SnoDRI. On the left-hand side is the flowchart with the steps followed in this study. On the right-hand side is the framework with autoencoder (top) and Mutual Information used to calculate the weight of each input variable. Inside the square bracket is the number of nodes in each layer of the autoencoder.

In this work, as shown in Fig. 2, we propose a novel index calculation framework. Here, we start with an available pool of meteorological variables, which might or might not be relevant to the drought type (snow droughts in our case). Firstly, the variables are filtered based on Random Forest regressions with available sets of observed variables, which can be indicators of droughts, as targets. Since we are interested in snow droughts and their hydrological impacts, we use SWE and discharge as target variables in the feature selection part of the framework (using Random Forest). It should be noted that a snow drought from the perspective of hydrological impacts cannot be equated to just a low SWE anomaly since several other meteorological factors would also influence the hydrological response. Based on the application, our framework allows the flexibility to assess any kind of drought stress by changing the potential indicator variables. After filtering variables, all of them were standardized up front, and the weight of each input variable was found from a self-supervised autoencoder model coupled with mutual information. Once the weights are obtained, each variable is multiplied with the corresponding weight, and the weighted inputs are added. Thus, the resultant values are standardized to obtain the SnoDRI index. Note that we utilize the initial 75% of the time series data, which pertains to the cooler months (October to May), to filter variables and determine their weights. The remaining data is reserved for validating the index, which is computed based on the filtered variables and their weights. In order to validate the index, we could find only limited observed records of potential indicators in the basin considered. This led us to validate the index based on SWE, SWE anomaly, snow fraction, temperature anomaly, precipitation, snow depth, and discharge. The details of the above-mentioned components of the framework are discussed in the subsequent sections.

#### Random forest regression

Random Forests are a collection of decision trees. Each tree in a Random Forest is built based on a random subset of variables from bootstrapped data, which can be used for classification and regression problems^42^. Since every regression tree inside the Random Forest identifies an order of variables to classify the training dataset, we can leverage the random forest regression algorithm to find the feature importance of training variables in predicting the target variable. In this study, we use Random Forest regression to select the important variables to develop SnoDRI. We aggregated the NLDAS-2 variables for 85 CAMELS basins in the Western US and regressed it against SWE and discharge within the basins. This yields two sets of feature importance corresponding to SWE and discharges for each basin. Then, by taking the mean of all basins, the average feature importance of variables for the Western US is calculated. The union of the top three variables in average feature importance for predicting SWE and discharge is used to compute SnoDRI. Note that both SWE and discharge variables are in effect only during the Random Forest feature selection. The idea here is to use available measured variables, which potentially indicate droughts, to filter variables accounted for the index calculation for the region. Once we obtain the filtered variables and their weights (discussed in a later section), they can be transferred to other ungauged basins in the region for calculating the index, which enables us to calculate the index for ungauged basins.

#### Self-supervised learning with autoencoder

Given the fact that we are trying to develop a novel snow drought index derived from different snow variables, we do not have a target variable to train a model. The absence of a target variable makes our problem a case of unsupervised learning. We used the ML algorithm of autoencoders, a particular type of neural network that is used for dimensionality reduction^43^. During the learning process, autoencoders “discard” the insignificant information present in the dataset. An autoencoder consists of an input layer, an encoder with hidden layers, a bottleneck layer, a decoder with hidden layers, and an output layer that tries to reconstruct the input data (self-supervised learning). The NLDAS-2 variables identified through Random Forest regression (Sect. “Random forest regression”) are initially passed to the input layer. As the data passes through the encoder and reaches the bottleneck layer, it encodes the entire data into a reduced form, which can be regarded as a ‘compressed’ form of important information in the entire dataset. After several trial-and-error iterations, we finalized an autoencoder network with three hidden layers, including a bottleneck. The structure of the autoencoder network is shown in Fig. 2. The bottleneck layer consists of one neuron, and the other two hidden layers consist of fifteen neurons. The hidden layers and the bottleneck layer used tanh activation functions, which take care of the nonlinearities in the model. We find that the Adam optimization algorithm with the Huber loss function gives better training of our model. The training of the model in 3000 epochs as single batches provided the best possible accuracy with the given datasets. Loss and accuracy stayed nearly the same regardless of the additional increase in the number of epochs. We use 75% of the entire dataset (those belonging to Oct-May) to train the autoencoder.

#### Mutual information as weights

Once the autoencoder is trained, we obtain the bottleneck output (or the encoder output) of the model, which is a single time series since the bottleneck consists of only one node. Our framework calculates the Mutual Information (MI) between the bottleneck output and each input variable, which is used as their weights (e.g., the weight of temperature equals the MI between bottleneck output and temperature). MI is a measure of how much information, on average, can one random variable tell us about another random variable^22,44,45^. It can be conceptualized as the reduction in entropy of one variable, given the information about another variable^21^. MI between two random variables, X and Y, expressed as I(X;Y), is calculated using Eq. (1)^22,44^.

1 $$I\left(X;Y\right)=\sum {P}_{XY}\left(x,y\right)log\frac{{P}_{XY}\left(x,y\right)}{{P}_{X}\left(x\right){P}_{Y}\left(y\right)}={E}_{{P}_{XY}}log\frac{{P}_{XY}}{{P}_{X}{P}_{Y}}$$

Here P_X_ (x) and P_Y_ (y) are marginal probabilities, and P_XY_(x,y) is the joint probability. As shown in Fig. 2, the weights of each variable are multiplied by themselves and added (weighted sum), which is then standardized to obtain SnoDRI values. Since the bottleneck represents a “compressed” version of all input data, the MI shows how much of each variable is contained inside the “compressed” form.

**Figure 3:**
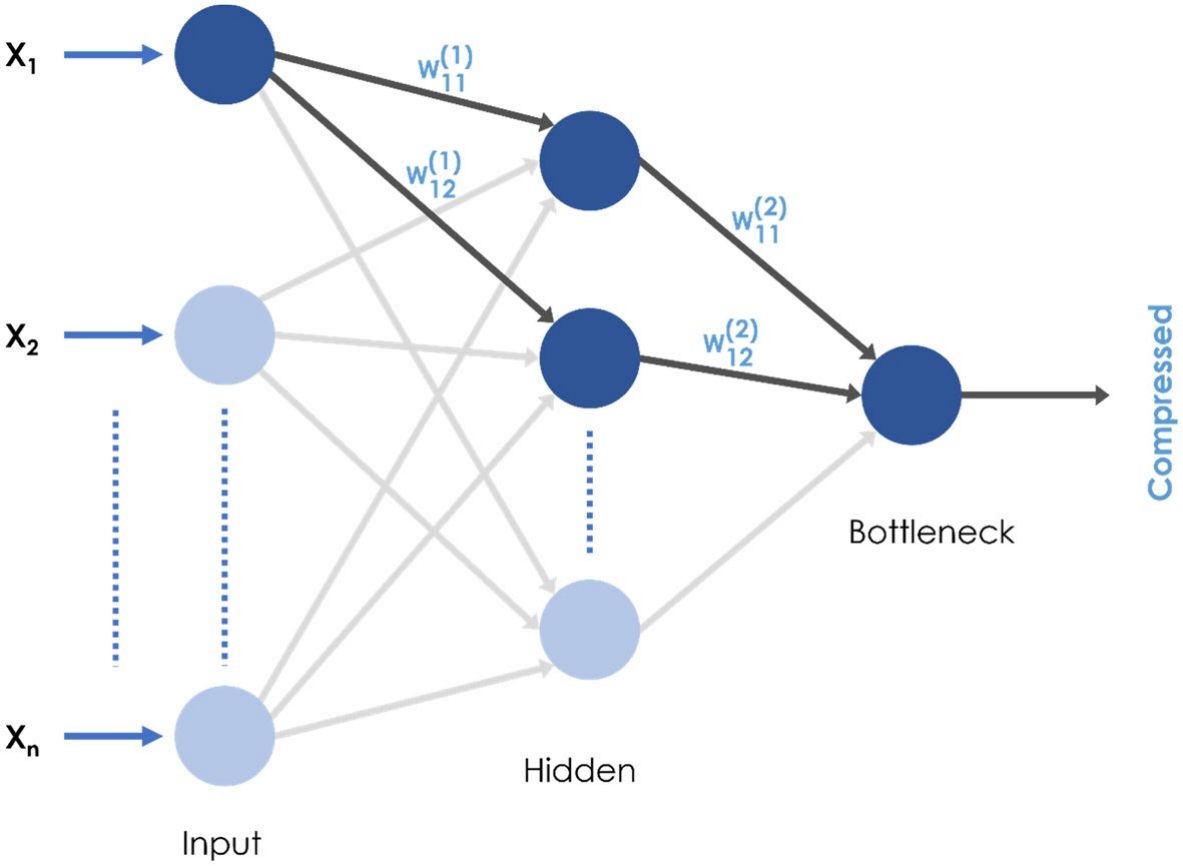
The encoder portion (first half) of the autoencoder neural network. The highlighted paths show two possible “information flows” (black arrowed lines) from the first input feature to the compressed bottleneck output.

A valid question here is why we cannot directly use the weights of the trained autoencoder. To explain this, referring to Fig. 3, we must look at all possible trajectories of “information flow” from one input variable to the compressed bottleneck output. We can see that other input variables also influence the weights. For example, the highlighted path in Fig. 3 shows possible trajectories of “information flow” from input X1 before it reaches the compressed bottleneck output. Since the nodes in the second layer are connected to all input nodes from previous layers, weights ${\text{W}}_{11}^{\text{(}\text{2)}}$ and ${\text{W}}_{12}^{(2)}$ are optimized based on the information from all input variables. As the information from each input node is divided and passed to all nodes in the hidden layer, weights ${\text{W}}_{11}^{(1)}$ and ${\text{W}}_{12}^{(1)}$ are optimized based on the division of information from input nodes. Therefore, the weights inside the autoencoder network are not representative of the relative contribution of each input to the compressed bottleneck output. This issue led to the use of an alternative method, i.e., MI, to infer the weights for each input feature.

#### Rain-snow partitioning

We also use snow fraction as an input into the model. In the modeling realm, there are different rain-snow partitioning schemes based on which precipitation variable is used as the forcing is separated into snow and rainfall. Classifying rain and snow based on a threshold temperature is the most straightforward scheme. This method is susceptible to the choice of threshold temperature. In another scheme proposed by Jordan (1991), the snow percent is calculated as a linear stepwise function of air temperature. In this study, we estimate the snow fraction based on a sigmoid function of wet bulb temperature, as Wang et al.^47^ proposed. The wet bulb temperature is calculated from air temperature, specific humidity, and surface pressure from the NLDAS-2 dataset.

#### Validation

There is no single framework for validating a drought index. Based on the purpose of the new index, one must examine whether the index follows the drought indicators, such as the scarcity of relevant environmental variables. This study uses the anomaly in SWE as an indicator variable. A negative anomaly represents the lack of snow accumulation compared to normal and vice versa. The lower the anomaly, the more severe the snow drought. The discharge co-occurring with SWE is another indicator variable. Reduced discharge due to low meltwater contribution can be a potential impact of the snow drought. A lower discharge following a lower SWE is a prime case of snow drought.

We compare the novel SnoDRI with patterns of SWE and discharge in the Upper Tuolumne basin of California. Hatchett et al.^8^ and Harpold and Dettinger^5^ reported a snow drought in this region during the winter (Jan to Apr) of 2014 and 2015. A lower SWE, along with a lower discharge, is taken as a signal of snow drought in the basin. We check if these signals correspond to a lower value of SnoDRI. Meanwhile, the index should not give a false positive forecast.

## Results

### Feature importance

**Figure 4:**
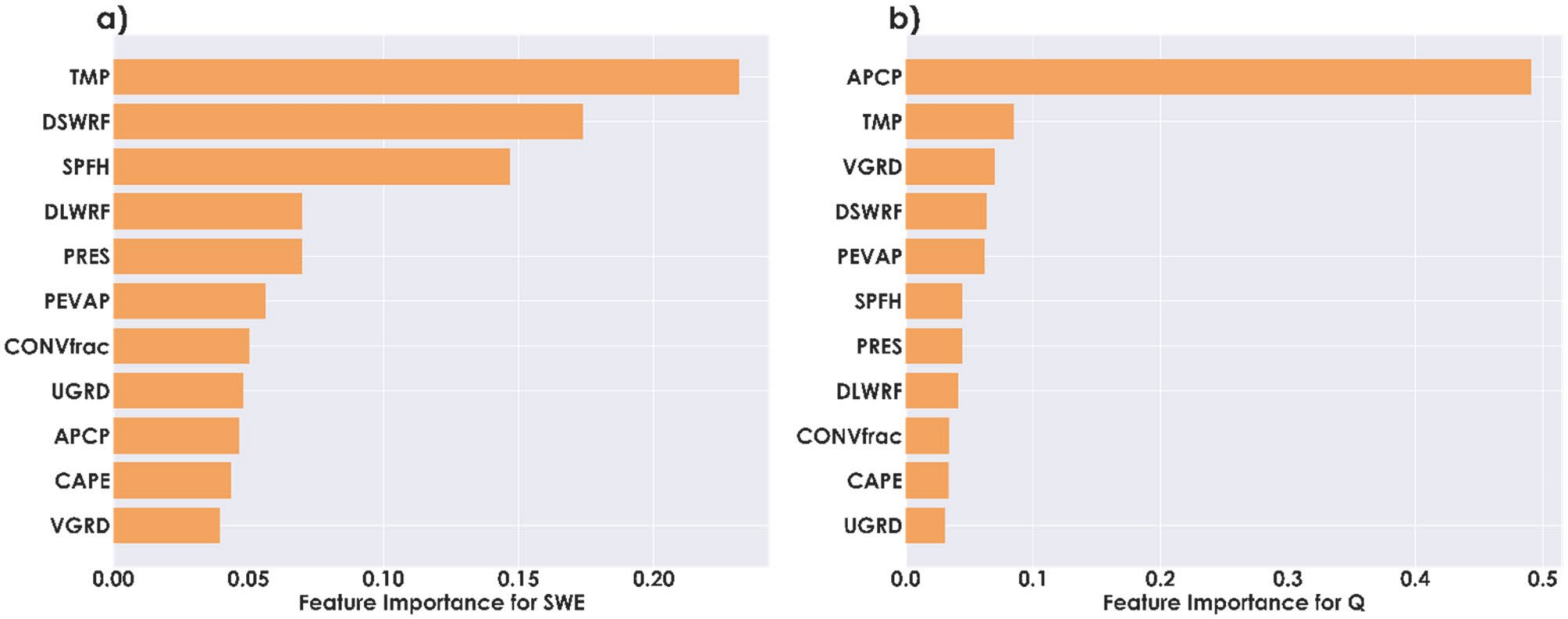
Average Feature importance for SWE (**a**) and Q (**b**) in basins of Pacific states. The long names of variables are provided in Table 1.

Through Random Forest regressions for 85 CAMELS basins, we obtained the average feature importance of NLDAS-2 variables for the Pacific Coast States of the US in predicting SWE and discharge. This method gives a sense of the relative significance of each variable in generating SWE or discharge. Figure 4a shows the feature importance for predicting SWE. We see that temperature is the top significant variable in determining SWE. This is most likely because the temperature decides the amount of snowfall (vs. rainfall) in the precipitation. Several rain-snow partitioning schemes are highly sensitive to temperatures^46–49^. After temperature, the downward shortwave radiation affects the SWE most. Since the primary source of energy that drives the atmospheric process is the incoming shortwave radiation, we can expect that the formation and accumulation of snow are highly dependent on shortwave radiation. Specific humidity is the third most crucial variable for SWE as obtained from Random Forest regression. Specific humidity (the measure of water content in the atmosphere) could have a significant effect on the formation of snow.

Figure 4b indicates the average feature importance for predicting discharge for the Pacific Coast States of the US. The results show that precipitation has a very high significance for estimating discharge in the basin. It is intuitive that the incoming precipitation, whether as snow, rain, or both, would contribute the most to generating river runoff as precipitation is the primary water source for any basin. This influence is pertinent when we consider cooler months separately, during which the precipitation occurs mainly as snow, which drives the snow accumulation and melting crucial for runoff generation. Though temperature, zonal wind, and downward shortwave radiation are most important after precipitation, their importance is far lower than precipitation, as the results of Random Forest regression suggest.

From both cases, Random Forest regression targeting SWE and discharge, we identified the top three variables having the highest average feature importance for our study area. In the case of SWE, the top three variables are TMP, DSWRF, and SPFH (Fig. 4a). Whereas for Q, the top three variables are APCP, TMP, and VGRD (Fig. 4b). The union of these two sets of variables gives a set of APCP, TMP, DSWRF, SPFH, and VGRD, which are used as input variables in SnoDRI calculations. Note that the union ended up with five variables since TMP is repeating in both sets.

### Weights from mutual information

**Figure 5:**
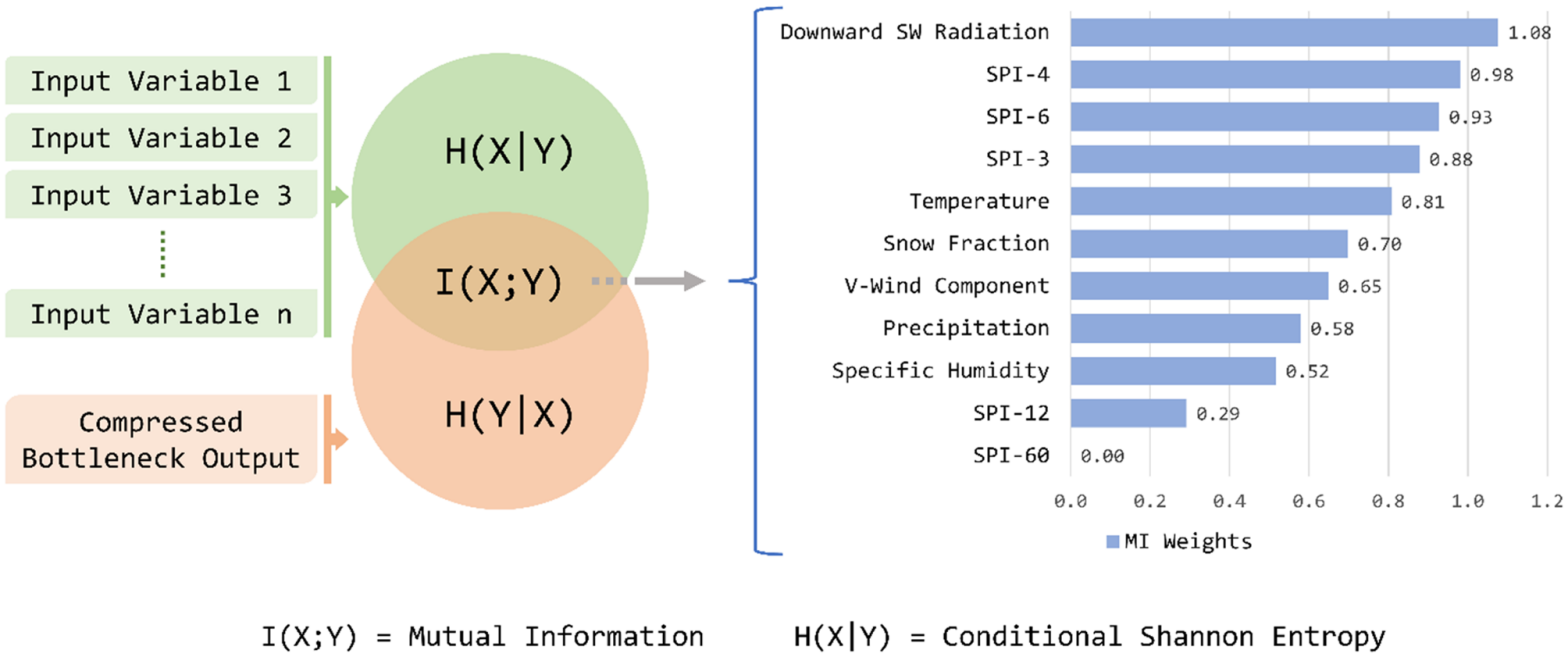
Weights obtained for each input variable as the Mutual Information in each variable and the compressed bottleneck output.

The MI between the compressed bottleneck output and each input variable measured the relative importance of the corresponding variable in the compressed data (bottleneck output). Figure 5 shows the approach for obtaining weights and the subsequent results. We can see that the downward shortwave radiation, SPIs, temperature, and snow fraction are found to be carrying more weight than other variables. Downward shortwave radiation from the sun entering the atmosphere, besides acting as the sole energy source of hydroclimatic processes, causes the direct melting of snow. This shows how important the downward shortwave radiation can be in the occurrence of snow droughts, which is reflected as the highest weight estimated by our framework. However, many of the previous studies on snow droughts have not considered the impact of downward shortwave radiation in their assessment^1,7,12^. Our result suggests further investigating the direct and indirect association between downward shortwave radiation and snow droughts. The model also identifies shorter-scale SPIs (3, 4, and 6 months) as significant. Since SPIs represent the lack of precipitation in the case of drought, they can be a proxy for lower snowfall and snowpack conditions. Following this, the model assigns more weight to temperature, complying with the fact that temperature drives snow processes from the formation of snow to the melting of snow. Snow fraction, another variable with considerable weight, can be directly related to the amount of snowpack. The lower the snow fraction, the lower the snowfall, leading to snow drought conditions. Interestingly, the model identifies that precipitation as such does not have a severe influence. The reason could be that the SPIs, which are the precipitation mapped to a normal distribution, already contain the information of precipitation. This can also be attributed to the ability of the model to dismiss redundant information. The lowermost weights are assigned to the SPI-12 and 60, two variables presupposed to be irrelevant for snow droughts, additionally confirming the aforementioned ability of the model.

### SnoDRI evaluation

**Figure 6:**
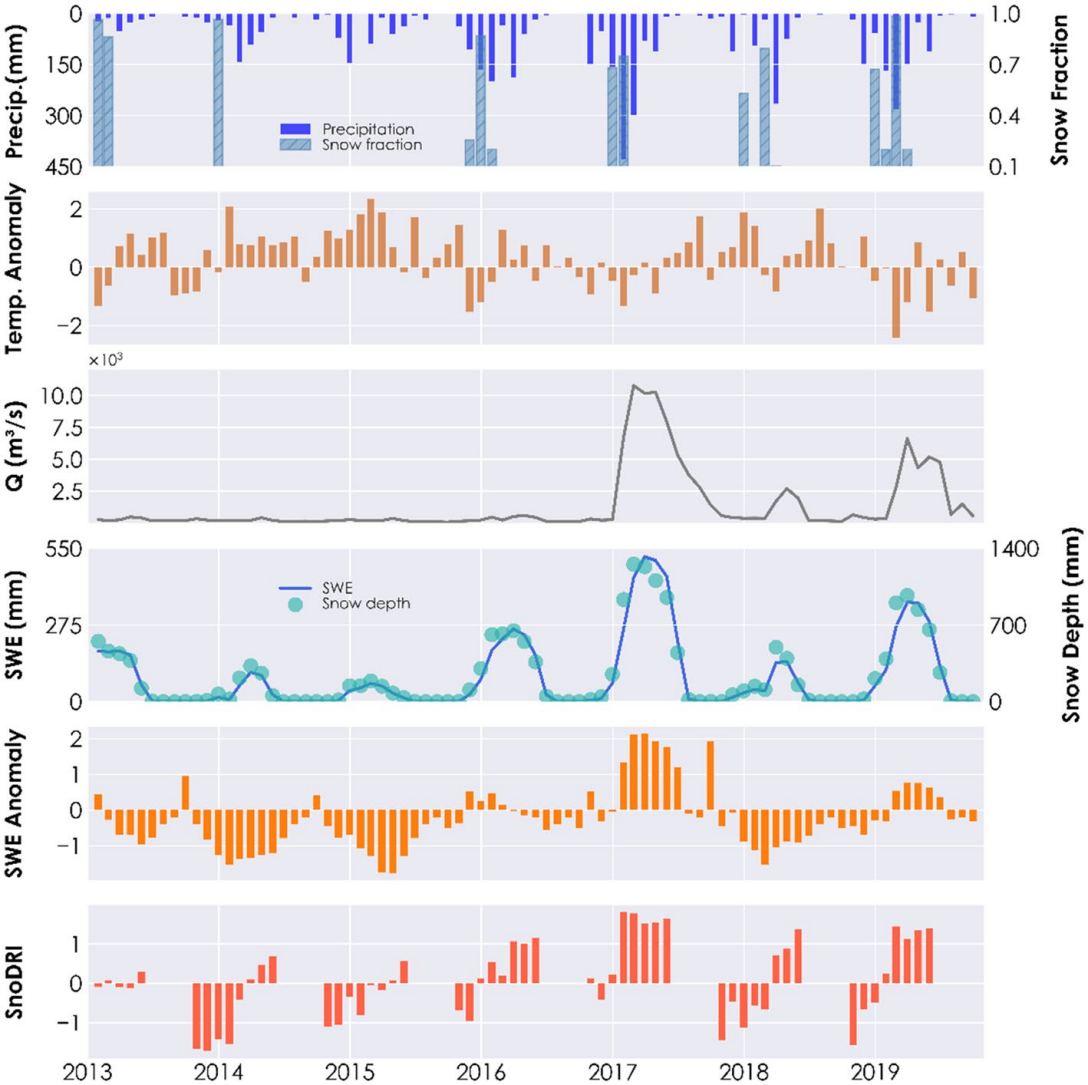
SnoDRI, SWE anomaly, SWE, and discharge in the Upper Tuolumne basin during the validation period.

With the newly developed snow drought index, SnoDRI, we analyzed the conditions of snow drought in the Upper Tuolumne River basin from February 2013 to May 2019. This period is included in the evaluation period. In other words, the model has never seen the input dataset of this duration throughout its training. Over this evaluation period, the new index shows a good performance in capturing snow drought events. Figure 6 shows the SnoDRI calculated for the Upper Tuolumne Basin in California, indicating a lower value corresponding to the reported snow droughts during the winter of 2013/14 and 2014/2015. Negative (positive) values of the SnoDRI suggest the presence (absence) and severity of the snow drought.

In addition, we place SnoDRI and the potential signals of the snow drought in juxtaposition. Comparing SnoDRI with the anomaly in SWE, SnoDRI shows lower (more negative) values when the SWE anomaly is shallow. For instance, in 2014, 2015, and 2018, the Upper Tuolumne Basin showed a negative anomaly in SWE (Fig. 6). During these years, SnoDRI shows lower values, indicating the occurrence of snow drought. On the other hand, in 2017, the region observed higher snow accumulation, leading to a positive SWE anomaly. SnoDRI indicates a continuous positive value during this period. Moreover, the higher (in 2017) and lower (in 2014, 2015, and 2018) values of original SWE and snow depth are reflected in SnoDRI. A lower discharge accompanied by lower SWE is another indicator of snow drought. In Fig. 6, during all winters from 2013 to 2019, except for 2017 and 2019, the Upper Tuolumne basin produced low discharges. SnoDRI identifies this signal. Whereas in 2017 and 2019, the basin received a higher snow accumulation, and as a result, the basin generated a higher discharge. This absence of snow drought is reflected in the SnoDRI, as seen in Fig. 6. The higher the temperature, the higher the chance of snow droughts, leading to reduced snowfall and rapid snowmelt. In accordance with Fig. 6, negative SnoDRI matches with positive anomalies in temperature and vice versa.

Given the ability of SnoDRI to capture snow droughts, we can implement the following steps to apply our new framework for a selected basin (in Pacific states) in a selected year: (1) obtain the filtered NLDAS-2 variables for the year of interest and aggregate them over the basin of interest, (2) calculate the SPIs and snow fraction, (3) derive the monthly time series and standardize them, (4) multiply the pre-calculated weights (see Fig. 5) with each variable and add them up, and (5) standardize the time series thus acquired.

## Discussion

Generally, this study proposes a new framework to calculate SnoDRI, an index that can measure snow droughts. The framework could be advantageous due to the multiple strengths that we identified. Firstly, it can be applied in ungauged basins. Since we use only the selected features from NLDAS-2 forcings, a gridded dataset integrating satellite observation with measurement gauges and radars, we do not need to rely on any ground-measured variables to calculate SnoDRI. Several basins worldwide are still ungauged, especially in developing countries, leading to a lack of an efficient drought monitoring system. Our framework can act as an alternative snow drought indication framework in such regions. Note that while NLDAS-2 integrates ground-based observations in their reanalysis process, it does not impede the applicability of the index over ungauged basins, as the gridded data extends over these areas. Secondly, our framework reduces the subjectivity in choosing the input variables by using Random Forest models to select important features. Previous studies examined snow droughts with definitions based on a handful of variables (mostly precipitation, temperature, and SWE) selected based on expert knowledge and assumptions, which add subjectivity to the analysis^1,12^. Thirdly, our framework, besides calculating the index, can give insights into what factors drive snow drought conditions, as represented by the weights from the autoencoder with MI. Typically, studies investigating snow droughts calculate the index based on the abnormal variations in snow variables. Fourthly, regardless of several model inputs that possess multicollinearity, a common issue while using multiple predictor variables, we can see that the SnoDRI framework could eliminate redundant information to a certain extent. For instance, the model gave a lower weight to specific humidity, a variable used along with temperature to calculate snow fraction. Finally, unlike SPI or Palmer Drought Severity Index (PDSI; Palmer, 1965^14^), the proposed ML-based framework is not sensitive to the time series length. Once the model is trained, its weights are fixed and can be applied to a new dataset, no matter the range of time. Moreover, since ML models are known for their capacity to learn complex patterns, we can relax the assumption of stationarity. However, the efficacy of SnoDRI in capturing multi-scale (both spatial and temporal) droughts needs to be explored in greater depth. The abovementioned capabilities of the framework highlight the competence of ML and information theory metrics in assessing snow droughts (or any drought, for that matter).

Formulating the framework for drought index calculation came with several challenges. Most importantly, the absence of a target variable to train and test the ML model. It is not straightforward to establish the performance of the index with a statistical metric (e.g., NSE or KGE). Rather, we had to compare and contrast the indicator variables with the index to see whether it was able to capture the signals of drought. This gave rise to another challenge: the lack of characterization of snow droughts in the present literature. Despite the ongoing interest in the topic, researchers have not reached an established definition of snow drought. Although our framework can overcome this issue since it is independent of a definition based on a single variable, more detailed investigation on snow drought characterization can better help assess their presence. Our framework, using the autoencoder, calculates the stress in the combined effect of hydrometeorological variables instead of looking at a single variable. However, validating this ability of the index can be tricky given the lack of understanding of the snow drought mechanism and the availability of observed data. This limits the validation process to be solely based on available records of indicator variables—another challenge in this study. It is a common problem in all drought index development studies^50^. Generally, the researchers compare a new index with some of the reported drought events to validate the index^51–53^. Nevertheless, this does not show exclusively the ability of an index to capture all the drought events. Some studies validate their index by checking its congruency with the US Drought Monitor^53,54^. There is vast room for research in developing an established framework for validating drought indices. A possible way to create a validation framework is to verify the closeness of the distributions of relevant variables with that of the index. The closeness of different distributions can be quantified with statistical methods.

We acknowledge that, to some extent, the index is susceptible to the group of input variables we start with before the Random Forest feature selection. The importance of any variable obtained from the Random Forest feature selection depends on the whole set of input variables. In other words, adding more variables or choosing a different dataset might produce a different order of feature importance. Several studies have employed a feature selection strategy based on sensitivity analysis (e.g., with normalized sensitivity coefficients)^55–57^. The effects of incorporating such methods, which could potentially further the feature selection component of the new framework, need to be explored. Although we only considered the NLDAS-2 variables in calculating SnoDRI, the framework can be applied to any set of time series input variables. For example, the ERA5-Land dataset, which provides a larger number of variables with a more extended period^58^, and the data from Land Information System (LIS) simulations. In spite of the high computational cost, it would be interesting to see the performance of the framework with ERA-5 or LIS variables. We have executed the framework at a basin scale in the western US. Future efforts can be directed towards establishing the framework in a gridded manner at continental or global scales.

Although we focus on snow droughts, this framework can be applied to identify any type of drought, given the appropriate input variables and feature selection. We select variables by regressing Random Forests against SWE and discharge. Training the Random Forests against different target variables relevant to the interested drought type would give another set of input variables. These can be transformed into an index by following the steps in our framework. Thus, by design, our framework is transferrable. We can set up the framework for any region of interest by training the Random Forest (for feature selection) and autoencoder (for estimating the weights of selected features through MI scores) with the data of that region. It should be noted that, for different regions, the model might assign different weights to variables depending on the hydroclimatic characteristics of the region. For instance, in the regions where temperature variability has greater influence, temperature is most likely to control the compressed bottleneck information inside the autoencoder, leading to a higher MI score for temperature (with bottleneck information). This aspect of our framework can be used to get insights about the impact of each variable on droughts.

## Conclusion

We developed a framework to calculate a new index, SnoDRI, that can be used to identify snow droughts. We trained Random Forest models for 85 basins across the West Coast to select the input features for the index calculation. Our novel framework showed the capability of combining autoencoders (a self-supervised machine-learning algorithm) with MI (a degree of mutual dependency between two variables) to estimate the importance of input variables in the occurrence of snow droughts. We found that the downward shortwave radiation, SPIs, temperature, and snow fraction considerably influence snow droughts. In validation, SnoDRI successfully captured the reported snow drought events and their signals in the Upper Tuolumne Basin in the Sierra Nevada region. The framework demonstrated the potential to eliminate redundant information in the dataset. The novelty of our framework is that it can be applied to ungauged basins since it does not use any ground measurements. The framework can be adapted to other types of droughts and to different regions around the world.

## Data Availability

The datasets used and/or analyzed in the study are available from the corresponding author on reasonable request.
